# Energy Expenditure Exceeds Nutritional Intake of ROTC Members During a Field Training Exercise

**DOI:** 10.3390/jfmk11010003

**Published:** 2025-12-23

**Authors:** Katherine A. Frick, Nicholas C. Bordonie, Katie G. Clouse, Michael D. Roberts, Andrew D. Frugé, Danielle D. Wadsworth, Matthew W. Miller, JoEllen M. Sefton

**Affiliations:** 1 Warrior Research Center, Auburn University, 301 Wire Rd, Auburn, AL 36832, USA; kaf0067@auburn.edu (K.A.F.);; 2 Nutrabolt Molecular and Applied Sciences Laboratory, Auburn University, 301 Wire Rd, Auburn, AL 36832, USA; 3 College of Nursing, Auburn University, 710 S Donahue Dr, Auburn, AL 36832, USA; 4 Exercise Adherence and Obesity Prevention Laboratory, Auburn University, 301 Wire Rd, Auburn, AL 36832, USA; 5 Performance and Exercise Psychophysiology Lab, Auburn University, 301 Wire Rd, Auburn, AL 36832, USA

**Keywords:** military nutrition, macronutrient intake, energy availability, caloric deficit

## Abstract

**Background:** Reserve Officer Training Corps (ROTC) Cadets undergo biannual Field Training Exercises (FTX) that impose substantial physiological demands, necessitating adequate nutritional intake to support performance and recovery. **Methods:** Energy Expenditure (EE) measured by actigraphy and self-reported nutritional intake (NI) of ROTC Cadets during a Fall FTX were obtained and compared to Military Dietary Reference Intake (MDRI) guidelines. Energy balance and nutrient adequacy were assessed using paired sample *t*-tests. **Results:** Cadets demonstrated significant caloric deficits, consuming fewer kilocalories than both their active metabolic rate (t = −12.07, df = 42, *p* < 0.001) and Low Energy Availability thresholds (t = 6.47, df = 57.54, *p* < 0.001). Macronutrient analysis revealed widespread deficiencies. Neither male nor female cadets met minimum carbohydrate gram recommendations. Protein intake in grams was significantly below MDRI guidelines for 94% of males (t = −10.03, *p* < 0.001) and 90% of females (t = −4.62, *p* = 0.001). Fat intake was generally adequate for all cadets, with 94% of males (t = 6.50, *p* < 0.001) and 90% of females (t = 4.19, *p* = 0.002) meeting or exceeding recommended fat intake. **Conclusions:** These findings underscore the prevalence of under-fueling during FTX and highlight the need for improved nutritional strategies to mitigate energy deficits and support cadet performance and health.

## 1. Introduction

Service members often engage in physically and mentally demanding tasks. Proper nutrition is essential for sustaining peak performance during these tasks [1,2]. The Military Dietary Reference Intake (MDRI) provides nutritional guidelines for military personnel based on training settings [3]. MDRI recommends normal daily caloric intake ranging from 1785 to 3417 kcal for males and 1432 to 2756 kcal for females. Macronutrient distribution should consist of 50–55% carbohydrates, 10–35% protein, and no more than 30% fat [3]. Micronutrient requirements are expressed as percentages of daily intake. An important training event for Reserve Officer Training Corps (ROTC) Cadets is the required twice yearly Field Training Exercise (FTX). During FTXs, Cadets primarily rely on Meal-Ready-to-Eat (MRE) rations provided by their training unit. MREs are designed to meet the nutritional demands of field training, offering high-calorie, nutrient-dense meals. However, food preferences, hunger levels, and meal timing often cause Cadets to self-limit their intake [4].

A negative energy balance of 30–45 kcal/kg/day increases the risk of decreased physical performance and impaired physiological function [5,6]. Insufficient nutritional intake combined with high activity levels has been linked to increased risk of bone stress injuries [7], menstrual and fertility disorders in women [5], and reduced testosterone levels in men [4,8,9]. Inadequate energy availability also affects cellular growth, maintenance, and thermoregulation [10]. Research has further demonstrated nutritional deficits during military training can impair physical and cognitive performance, endocrine and metabolic function, gastrointestinal health, iron status, mood, and immune function [11].

Military nutritional needs are often compared to those of athletes, and key differences exist. Variations in training intensity, duration, and personnel demographics influence nutritional demands. Military personnel often require higher fat intake to support nutrient absorption and overall caloric needs [6,12]. Adequate fat in the military members’ diet also supports prolonged tasks, such as an FTX. Athletic nutrition recommendations typically establish intake based on body composition and focus on the desired performance need of the athlete based on sport, position, and activity requirements [4,13]. In contrast, military nutritional recommendations are developed around percentages for macronutrients and recommended in terms of daily grams of macronutrient per kilograms of body weight [3]. These macronutrient per kilogram body weight recommendations are altered during operational and restricted rations [3].

The United States ROTC units conduct biannual FTXs lasting two to three days. These exercises involve military training activities such as marksmanship, obstacle courses, land navigation, and ruck marches [14]. Cadets must complete training while living in field conditions requiring them to adapt their nutrition to meet physical demands [15]. Proper nutritional intake reduces fatigue, injury risk, and illness [16]. However, studies indicate that military members often experience a nutritional deficit compared to their energy expenditure during multi-day exercises [4,6,17,18]. This study aims to: (a) assess self-reported nutritional intake (NI) among ROTC Cadets during a short-term FTX compared to military nutrition guidelines; and (b) compare self-reported NI with calculated energy expenditure (EE) during the FTX.

## 2. Materials and Methods

**Figure 1:**
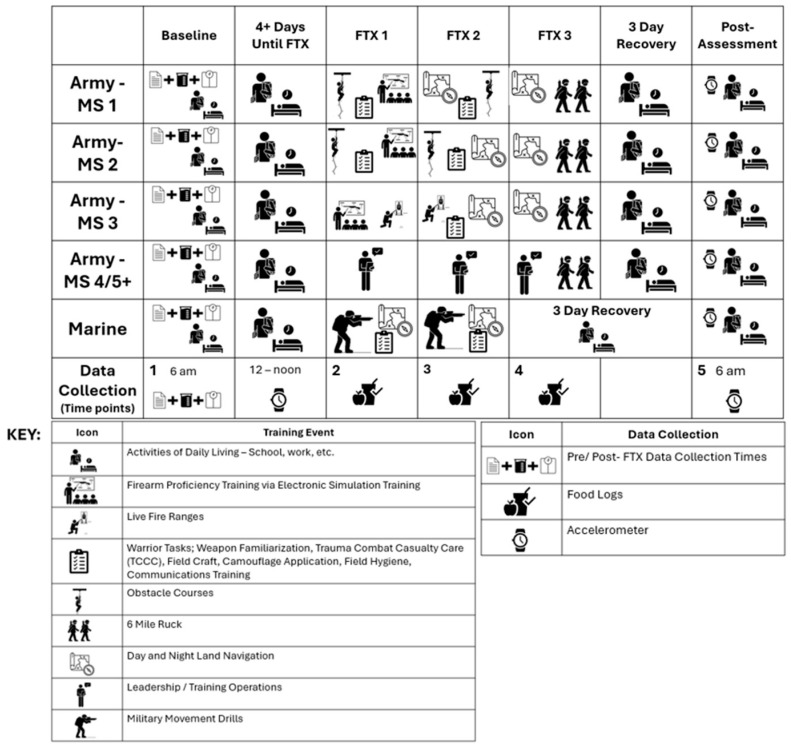
Field Exercise Training (FTX) and Data Collection Time Point Schedule.

This study was part of a larger observational cohort study conducted with a university ROTC program. The university’s Institutional Review Board, Army and Marine ROTC Commands and Army HPRO approved the study (protocol #24-843 FB). Participants wore accelerometers to monitor activity levels and self-reported their estimated nutritional intake during a two-to-three-day FTX. The training program, duration, meal availability, and physical demands varied by ROTC branch (Army or Marine) (Figure 1).

### 2.1. Field Exercise Training (FTX) Protocols by Military Branch

**Marine ROTC Protocol:**

- Duration: Two days, one night of continuous training.
- Activities: Squad movement drills and patrol, bivouac infiltration and exfiltration, camouflage application, weapon system familiarization, communication system use, trauma combat casualty care (TCCC), day and night land navigation, logistics operations, leadership exercises, and ruck marches between activities.

**Army ROTC Protocol:**

- Duration: Three days, two nights with designated six-hour sleep periods per night.
- Activities varied by Military Science (MS) year:

  ○ **MS1 and MS2:** Firearm proficiency training via Electronic Simulation Training (EST), obstacle course, conditioning course, team development course, and a six-mile ruck march.
  ○ **MS3:** Firearm proficiency training via EST, live firearm training, and qualification at 25 m and modified record fire ranges.
  ○ **MS1–MS3:** Day and night land navigation, team-building events, warrior tasks (weapon familiarization, TCCC, field craft, camouflage application, field hygiene, and communications training).
  ○ **MS4:** Operational planning and support for MS1-MS3 events.

Participants supplemented MREs with self-provided food and beverages. All additional intake was recorded alongside military-provided meals.

### 2.2. Participants and Participant Demographics

All participants voluntarily joined this study and signed a written informed consent after receiving a detailed verbal and written explanation of the requirements. To be eligible, individuals had to be active members of the university ROTC program, between 17 and 45 years old, free of physical restrictions, not on a medical profile or chit, and without known medical conditions that would disqualify them from Department of Defense service.

**Figure 2:**
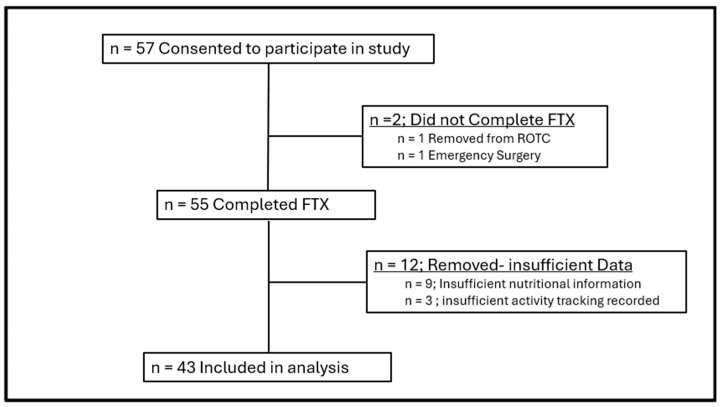
Participant flow and sample size.

Fifty-seven individuals initially enrolled in the study. Fourteen participants were later excluded from the analysis due to incomplete data or other complications (Figure 2). The final analysis included 43 participants (33 males: 20.39 ± 3.37 years, 175.96 ± 5.72 cm, 79.65 ± 8.61 kg; 10 females: 23.30 ± 6.5 years, 166.68 ± 5.17 cm, 67.75 ± 7.92 kg). Additional demographic details are presented in Table 1.

### 2.3. Participant Data Collection

All volunteers for this study completed the informed consent procedures with a research team member prior to beginning the baseline screening process. Baseline screening was completed up to three weeks prior to the FTX. Participants completed a health history and demographics form.

Basic anthropometric assessments were conducted during the baseline visit. Participant height was recorded using a stadiometer (SECA, Hamburg, Germany). Weight and body composition were assessed using the TANITA BC-568 InnerScan Segmental Body Composition Monitor (TANITA Corporation, Preston, WA, USA), bioelectrical impedance technology. Sufficient hydration (USG ≤ 1.030) was ensured prior to measurement. Weight (lbs)/mass (kgs), lean muscle mass (kgs), body fat percentage, and visceral fat rating were recorded.

Participants recorded all nutritional intake from meals and snacks using a written food log. The log included pre-printed Meal-Ready-To-Eat (MRE) check sheet with additional space for documenting extra food items and portion sizes. The Army ROTC unit received two MREs per day during the FTX and one hot meal (HOT-A) in the field from the Army Post dining facility. The Marine ROTC unit received two or three MREs in advance and had the option to fieldstrip the MREs by discarding any unwanted items before the training event to minimize load packed in/out.

Caloric values for recorded food items were calculated using nutritional data from the Combat Rations Database (ComRAD), a resource developed by Human Performance Resources (Consortium for Health and Military Performance, CHAMP), the U.S. Army Combat Capabilities Development Command (DEVCOM) Soldier Center, and the U.S. Army Research Institute of Environmental Medicine (USARIEM) [19]. Additional food items were analyzed using the U.S. Department of Agriculture’s Food Data Central [20]. Because the Marine FTX lasted two continuous days while the Army FTX spanned three days, reported nutritional values were adjusted to reflect daily intake for direct comparison.

Energy expenditure was estimated using a triaxial accelerometer (ActiGraph xGT3X-BT, ActiGraph Technologies, Pensacola, FL, USA). Accelerometers were set to a 60 Hz sampling frequency and programmed for each participant with sex, age, height, body mass, and hand dominance. Participants wore the devices continuously throughout the FTX on the wrist with a secure strap. Raw acceleration data were analyzed using ActiLife Software v6.13.4 (ActiGraph, Pensacola, FL, USA). Energy expenditure was determined using built-in algorithms, including the Freedson VM3 Combination (2011) and Swartz Adult Overground & Lifestyle (2000) for metabolic equivalents (METs) [21]. Data were excluded from analysis if participants wore the device for less than 10 h per day.

Estimated energy balance (EB) was calculated by comparing energy expenditure (EE) with recorded nutritional intake (NI). A surplus of intake was recorded as a positive value, while a deficit was recorded as a negative value, following the equation:

E(nergy) B(alance) = N(utritional) I(ntake) − E(nergy) E(xpenditure)     or EB = NI − EE

This calculation was applied to each ROTC participant to assess daily energy balance.

Low Energy Availability (LEA) refers to an imbalance between NI and EE, or a negative EB, during an activity or exercise [8,22]. This imbalance results in insufficient energy to meet the body’s overall physiological demands. If an individual consumes less calories than they burn they fall into a LEA state in which the body lacks adequate energy to sustain optimal health and performance [11,22,23,24]. This low energy consumption state exists along a continuum of severity ranging from mild or adaptive conditions with minimal consequences to severe cases that may cause significant and potentially long-term impairments in health and overall physical performance [22]. Previous work has indicated that individuals can withstand LEA until a threshold is surpassed [22,25]. The caloric consumption measures for individuals are calculated by assessing fat free mass (FFM) rather than overall body weight [10,22]. The International Olympic Committee provides LEA standards as males consuming less than 9–25 kcal/kg/FFM/day and females consuming less than 30 kcal/kg/FFM/day [10]. For this study, we applied individual calculations for males quantifying LEA at <25 kcal/kg/FFM/day.

### 2.4. Statistical Analysis

Statistical analyses were performed using R Statistical Program Software Version 4.2.2 (RStudio; Boston, MA, USA) with the psych 2.3.3, lattice 0.20.45, dplyr 1.1.2, tidyr 1.2.0, emmeans 1.8.7, and effsize 0.8.1 packages. Statistical significance was set a priori at α = 0.05.

Assumption testing included assessment of normality using Shapiro–Wilk tests and visual inspection of Q-Q plots, and homogeneity of variances using Levene’s test. When assumptions of normality were met, parametric tests were applied; otherwise, equivalent nonparametric procedures were utilized.

One-sample *t*-tests, with corresponding Cohen’s *d* effect sizes, were conducted using the effsize R package and 95% confidence intervals to compare the participant’s total energy and macronutrient intake to MDRI recommendations. Separate analyses were conducted for males and females, with population means (µ) defined as MDRI recommended intakes expressed in grams of total nutritional intake. Paired *t*-tests or Wilcoxon signed-rank tests, as appropriate, evaluated differences between energy intake and energy expenditure to assess daily and overall energy balance during the FTX.

## 3. Results

### 3.1. Energy Expenditure and Energy Intake

During the FTX, most cadets did not meet the minimum caloric intake recommended by the MDRI. Mean total energy intake was 1120 ± 486 kcal for male cadets and 970 ± 422 kcal for female cadets. Only 12% of male cadets (n = 4) and 10% of female cadets (n = 1) consumed at least the MDRI minimum for total energy intake. When examined by military branch, 14% of male Army cadets (n = 4) and 20% of female Army cadets (n = 1) met the minimum recommended caloric intake, whereas no male or female Marine cadets (0%) achieved the minimum MDRI standards.

**Figure 3:**
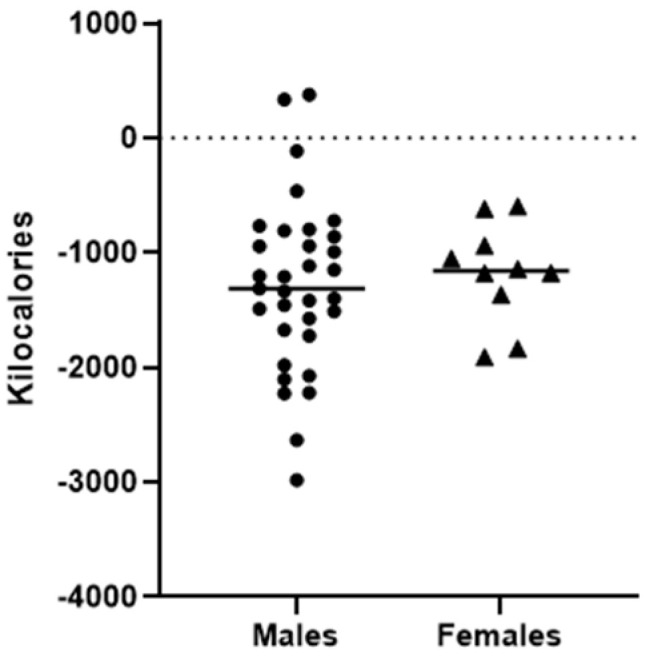
Energy Balance in kilocalories. Note: Calculation of Energy Balance (Nutritional Intake—Energy Expenditure); Male average (−1350 ± 674 kcal), Female Average (−975 ± 671 kcal).

The imbalance between Energy Expenditure and Nutritional Intake during the FTX resulted in a negative Energy Balance among both male and female cadets regardless of military branch (Figure 3). On average, male cadets demonstrated an energy deficit of −1350 ± 674 kcal, while female cadets exhibited a deficit of −975 ± 671 kcal. These findings indicated that the total EE substantially exceeded NI, contributing to negative EB and LEA during the FTX (Figure 3).

### 3.2. Macronutrient Consumption and Distribution

**Figure 4:**
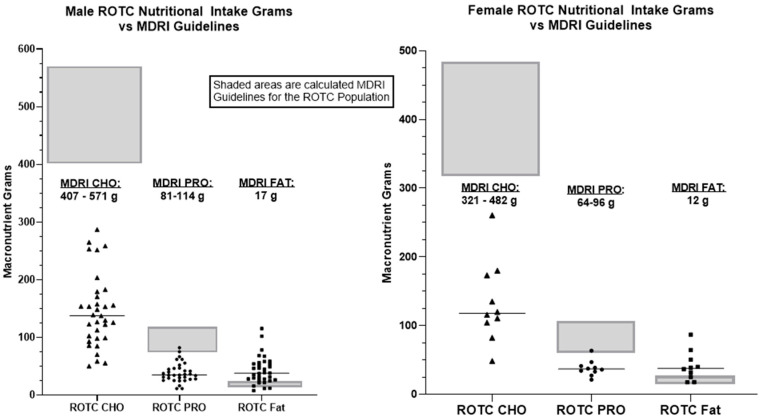
ROTC participant Nutritional Intake compared to MDRI quantities. Note: CHO = Carbohydrates, PRO = Protein; Male average: CHO: 145 g, PRO: 39 g, Fat: 43 g; Female average: CHO: 133 g, PRO: 38 g, Fat: 41 g. Triangles are CHO, circles are PRO, and Squares are FAT.

Comparison of recorded nutritional intake to MDRI guidelines, assessed using one sample *t*-tests with population means (µ), revealed substantial nutritional deficits and poor macronutrient distribution among ROTC cadets participating in the Fall FTX (Table 2, Figure 4). MDRI guidelines provide a recommended macronutrient range both in grams per day, and in percentage of daily nutritional intake. For example, carbohydrates should compose 50–55% daily nutritional intake, recommendations are from 340 g to 680 g per day for males, 276 g to 552 g for females. These recommendations will be referred to as the minimum recommendation and daily recommendations based on biological sex.

Table 2 presents a comparison between the MDRI recommendations and the recorded nutritional intake of Cadets during the FTX. Both males and females fell below MDRI ranges resulting a substantially lower daily caloric intake than recommended. When recorded nutritional intake was compared with the estimated energy expenditure during the FTX, the overall deficits fell below individually calculated LEA, with males averaging a deficit of approximately 2981 kcal and females with an approximate deficit of 2228 kcal per day.

Figure 4 illustrates the discrepancy between the nutritional intake of the ROTC Cadets compared to the MDRI macronutrient guidelines. Cadets consumed markedly less carbohydrate and protein than recommended as indicated by the shaded box regions on each graph in the figure. This pattern is consistent for box male and female Cadets, with carbohydrate intake showing the greatest deviation from the MDRI. The tight clustering of the individual data points across the cohort emphasizes the uniformity of overall caloric underconsumption of the ROTC Cadets. Overall, Figure 4 highlights the substantial insufficiency in macronutrient intake during the FTX relative to operational nutritional standards. This reinforces concerns of adequate nutritional intake during field training activities.

The MDRI provides a single fat intake recommendation based on biological sex. Most male cadets (94%) met or exceeded the daily fat gram recommendation (t = 6.50, df = 32, *p* < 0.001). Among female cadets, 90% met or exceeded the daily fat gram recommendation (t = 4.19, df = 9, *p* = 0.002).

All male cadets (t = −17.76, df = 32, *p* < 0.001) and female cadets (t = −22.37, df = 9, *p* < 0.001) failed to meet the minimum daily MDRI suggested grams of carbohydrates.

Protein intake analyses revealed 94% of male cadets did not meet the minimum daily recommended grams of protein (t = −10.03, df = 32, *p* < 0.001) and no male cadets exceeded the upper MDRI protein recommendation (t = −34.70, df = 32, *p* < 0.001). Only 10% of female cadets met the minimum MDRI grams of protein recommendation (t = −4.62, df = 9, *p* = 0.001) and none exceeded the daily recommendation (t = −16.56, df = 9, *p* < 0.001). (Figure 4).

## 4. Discussion

This study examined the self-reported nutritional intake and estimated energy expenditure of ROTC cadets during a Fall FTX. The goal was to provide insights into the balance between intake and expenditure under demanding field training conditions. The findings highlight the risk of energy deficits among cadets, which can significantly impact physical performance, cognitive function, injury risk, and overall health [4].

Energy balance depends on multiple factors, including assigned duties, task duration, environment, and food availability [26]. Poor energy balance, especially in a state of LEA where the body does not have the energy to support physiological functions, is associated with negative health outcomes including impaired immune and endocrine function, decreased bone mass, reduced strength and power, and unfavorable changes in body composition that can lower operational performance [11]. Previous research found soldiers expended approximately 595 more kcal per day than they consumed during the initial two weeks of basic training [18]. A 500 kcal daily deficit may not pose immediate health risks, but prolonged energy imbalances can negatively affect body composition and performance as well as mental and physical recovery. Severe energy depletion (e.g., 5% body fat loss or over 10% total weight loss) has been shown to impair health and job performance in military personnel [27,28,29].

Energy balance plays a crucial role in sustaining cognitive function, endurance, reaction time, and overall physical performance, all essential for military effectiveness [30]. A negative energy balance like the one observed in the current study can increase fatigue, impair decision making, and reduce muscular endurance [26,28,31,32]. These deficits may limit a cadet’s ability to perform effectively during training and in real-world operations. Research has shown that prolonged energy deficits hinder mission success, especially in environments where soldiers operated for extended periods with limited nutritional intake [33,34].

Cadets in the present study exhibited a statistically significant negative energy balance throughout the FTX (Figure 3). Research suggests energy deficits in training environments can contribute to LEA even in short-term settings such as a two-to-three-day FTX. Energy deficits were similar between ROTC branches despite differences in food provisions or scheduled activities. Army ROTC cadets received scheduled mealtimes and were provided with two MREs and one hot meal per day, while Marine ROTC cadets received two to three MREs before the event and were instructed to field strip non-essential items before training, allowing the Marines to carry less weight and use less space for food in their rucksacks.

LEA presents a significant challenge for military personnel during FTX where prolonged physical demands often coincide with insufficient caloric intake. Sustained LEA can impair performance, delay recovery, and hinder cognitive function, all of which are critical for mission success [18,23,29,35,36]. Chronic LEA can also lead to relative energy deficiency in sport (RED-S), a condition characterized by compromised health and performance [10,23]. Most cadets in the present study not only failed to meet recommended caloric intake (Figure 3), but also fell below the IOC’s LEA threshold defined as <30 kcal/kg fat-free mass (FFM) per day for females and <25 kcal/kg FFM/day for males (Figure 4) [10]. These findings emphasize the need for proper energy balance to support health and performance during training. Energy deficits during training may also have long-term consequences for the health and career longevity of cadets. Chronic LEA increases the risk of stress fracture, muscle loss, and endocrine dysfunction which can lead to limited military performance and retention [11,23,24]. The findings in the current study suggest that early-stage training environments may predispose individuals to physiological adaptations that negatively impact performance.

Adequate energy intake is fundamental to achieving appropriate macronutrient distribution and supporting optimal physiological function, performance, and recovery. In this study, cadets exhibited notable caloric shortfalls that contributed to insufficient carbohydrate and protein consumption. Carbohydrates serve as the primary substrate for high-intensity and endurance activities, with recommended intakes ranging from 3–12 g/kg/day for athletes and 4–8 g/kg/day for military personnel [31,37]. Cadets, however, consumed only 142 ± 63 g/day (53 ± 8% of total energy), and none met established recommendations. These values are considerably lower than those reported in Initial Entry Training (IET) soldiers, who consumed approximately 240 g/day [17] or 5 g/kg/day [18]. Similarly, protein intake, which is essential for muscle maintenance, recovery, and training adaptation, was markedly below recommended ranges of 0.8–2.2 g/kg/day [1,37,38], with 94% of males and 90% of females failing to meet the minimum threshold. These deficits likely reflect overall energy insufficiency rather than selective macronutrient restriction, as reliance on high-fat, energy dense snack items (e.g., jerky, nut butters) may have promoted satiety and displaced carbohydrate and protein rich foods. Collectively, these findings indicate a pervasive energy imbalance that limits substrate availability for both immediate performance and adaptive processes, underscoring the importance of targeted nutritional strategies, such as carbohydrate and protein preloading, to enhance energy availability and support performance demands during field training and operational contexts.

Previous research has reported military personnel expending 3700–6300 kcal/day in training environments [39] and 3441.6 kcal/day in garrison [40]. Energy expenditure varies based on occupational tasks, with infantry soldiers often exhibiting the highest demands. Reported energy requirements range from 2342 kcal/day for female administrative personnel [33] to 7122 kcal/day for male Marines in mountain warfare training [29,41]. In this study, male cadets expended 2540.8 ± 638.9 kcal/day, while female cadets expended 1942.67 ± 368.58 kcal/day, both lower than previous literature [1,2,15,18,30]. This may be due to several factors including variations in activity level, training intensity, the shorter duration of this FTX compared to longer military exercises, or the methodology of estimating energy expenditure.

A variety of practical recommendations can be made to address the energy deficits observed in this study. Previous research has shown that providing protein shakes can increase caloric intake and enhance performance in Army Basic Combat Training [42]. Encouraging cadets to carry nutrient-dense lightweight snacks such as energy gels or carbohydrate-dense meal replacements may also help maintain caloric intake without increasing ruck sack weight or space [2,43]. Implementing structured meal planning before field exercises including carbohydrate-loading strategies may enhance energy availability. Teaching cadets military-specific nutrition strategies can empower them to make better fueling decisions, especially when in rationed environments [44,45]. Finally, encouraging optimized post-event nutrition including protein and carbohydrate replenishment may help in accelerating recovery and improving subsequent training performance. The purpose of the ROTC is to create future military leaders. It is important for ROTC cadets to learn and establish proper nutrition practices in these short-term, controlled training environments to set them up for success after commissioning. Integrating nutrition education into ROTC programs could help cadets develop effective fueling strategies before they enter active-duty service, enhancing resilience and improving long-term operational readiness.

This study has several limitations that should be considered when interpreting the findings. First, dietary intake was assessed using self-reported methods, which are prone to recall bias and underreporting, particularly in field training environments where accurate tracking is challenging. Additionally, energy expenditure was estimated using accelerometry, which, while practical and validated in military settings, does not account for load carriage or effort variations, potentially leading to underestimations. Prior research supports accelerometers as a valid tool for assessing physical activity and energy expenditure, particularly in military settings [6,18,46,47,48,49]. Additionally, accelerometers offer a non-invasive alternative that improves participant adherence without adding unnecessary burden [36,50,51,52]. Accelerometers measure acceleration; however, they do not directly measure movement effort. Heart rate variability over the course of an FTX would provide a more insightful measure into the true effort of the cadets. A difference in energy expenditure and effort is likely to occur when comparing a six-mile walk to a six-mile ruck with an additional 11.5 kgs (25 lbs) of gear and equipment. Measuring heart rate variability would provide a more insightful assessment of the effort that cadets are withstanding rather than just the distance. The study was also limited by its short duration, capturing only a snapshot of energy balance during a single FTX, which may not fully represent long-term patterns or cumulative effects of energy deficits. Furthermore, while differences in ROTC branch provisions were noted, individual variations in food consumption and adherence to nutritional recommendations were not controlled.

## 5. Conclusions

This study highlights observed energy deficits during FTX, underscoring the need for strategic fueling strategies, improved ration compositions, and enhanced nutrition education to sustain both training and operational performance. ROTC cadets may fail to consume sufficient nutrition to maintain energy balance, with dietary choices leading to excessive fat intake while falling short on carbohydrates and protein. The energy demands of FTX exceeds caloric intake, resulting in short-term LEA, which may impact performance and increase injury risk. Integrating sports nutrition principles and tactical meal planning into military training programs can help optimize energy balance, enhance mission readiness, and improve long-term force sustainability. Future research should further investigate the effects of LEA on military readiness and develop targeted nutritional strategies to sustain performance and resilience.

## Figures and Tables

**Table 1:** Participant Demographics.

Participants	43
MS1	11 (10 male/1 female)
MS2	11 (11 male/0 female)
MS3	8 (5 male/3 female)
MS4	11 (6 male/5 female)
MS5+	2 (1 male/1 female)
Male/Female	33/10
Age (years)	21 ± 4 years
Height (cm)	174 ± 7 cm
Body mass (kg)	76.9 ± 9.8 kg
BMI	25.4 ± 2.6
Body Fat Percentage (group)	20.2 ± 7.5%
Male Body Fat Percentage	17.0 ± 5%
Female Body Fat Percentage	31.3 ± 3.4%

Note: MS = Military Science Year.

**Table 2:** Macronutrient Distributions; Comparison of MDRI Guidelines and Cadet FTX Nutritional Intake.

	Males			Females		
	g	%	kcal	g	%	kcal
**MDRI**						
CHO	340–680 g	50–55%	1360–2720 kcal	275–552 g	50–55%	1100–2208 kcal
PRO	68–136 g	10–35%	272–544 kcal	55–110 g	10–35%	220–440 kcal
FAT	17 g	>30%	153 kcal	12 g	>30%	108 kcal
total kcal			1785–3417 kcal			1428–2756 kcal
**FTX Nutritional Intake**						
CHO	51–287 g	35–67%	204–1148 kcal	48–261 g	40–65%	192–1044 kcal
PRO	12–83 g	9–24%	48–332 kcal	12–62 g	10–20%	48–248 kcal
FAT	8–116 g	20–53%	72–1044 kcal	12–59 g	22–44%	108–531 kcal
total kcal			350–2361 kcal			336–1792 kcal
**FTX Energy Expenditure**			1622–3595 kcal			1417–2727 kcal
**LEA–Calculated**			1202–2063 kcal			1165–1586 kcal
**FTX Energy Balance**			(−2981)–(+334) kcal			(−2228)–(+375) kcal

Note: g = grams, % = percentage, kcal = kilocalories, MDRI = Military Dietary Reference Intake, CHO = Carbohydrates, PRO= Proteins, FAT = Fats, FTX = Field Training Exercises, LEA = Low Energy Availability (calculated for Cadets based on International Olympic Committee Standards).

## Data Availability

The data presented in this study are available on request from the corresponding author due to the potential of the ROTC members joining the United States military.

## Abbreviations

The following abbreviations are used in this manuscript:

ROTC	Reserve Officers’ Training Corps
FTX	Field Training Exercise
NI	Nutritional Intake
MDRI	Military Dietary Reference Intake
EE	Energy Expenditure
MRE	Meal-Ready-to-Eat
Kcal	Kilocalorie
Kg	Kilogram
TCCC	Trauma Combat Casualty Care
MS	Military Science
EST	Electronic Simulation Training
Cm	Centimeters
%	Percent
ComRad	Combat Rations Database
CHAMP	Consortium for Health and Military Performance
METs	Metabolic Equivalents
MVPA	Moderate-to-Vigorous-Physical-Activity
EB	Energy Balance
AMR	Active Metabolic Rate
IOC	International Olympic Committee
LEA	Low Energy Availability
RED-S	Relative Energy Deficiency in Sport
FFM	Fat Free Mass
IET	Initial Entry Training
g	Grams
CHO	Carbohydrates
PRO	Protein
Lbs	Pounds
